# Disease Modification in Multiple Sclerosis by Flupirtine—Results of a Randomized Placebo Controlled Phase II Trial

**DOI:** 10.3389/fneur.2018.00842

**Published:** 2018-10-09

**Authors:** Jan Dörr, Klaus-Dieter Wernecke, Jens Würfel, Judith Bellmann-Strobl, Volker Siffrin, Muriel B. Sättler, Mikael Simons, Andreas Linsa, Hayrettin Tumani, Friedemann Paul

**Affiliations:** ^1^NeuroCure Clinical Research Center, Charité – Universitätsmedizin Berlin, Corporate Member of Freie Universität Berlin, Humboldt-Universität zu Berlin, Berlin Institute of Health, Berlin, Germany; ^2^Charité – Universitätsmedizin Berlin and SOSTANA GmbH, Berlin, Germany; ^3^MIAC AG, Department Biomedical Engineering, University Basel, Basel, Switzerland; ^4^Experimental and Clinical Research Center, Max Delbrueck Center for Molecular Medicine and Charité – Universitätsmedizin Berlin, Corporate Member of Freie Universität Berlin, Humboldt-Universität zu Berlin, Berlin Institute of Health, Berlin, Germany; ^5^Neurozentrum Offenburg, Offenburg, Germany; ^6^Institute of Neuronal Cell Biology, Technical University Munich, Munich, Germany; ^7^German Center for Neurodegenerative Disease (DZNE), Munich, Germany; ^8^Munich Cluster for Systems Neurology (SyNergy), Munich, Germany; ^9^Klinik für Neurologie, Carl-Thiem Klinikum Cottbus gGmbH, Cottbus, Germany; ^10^Klinik für Neurologie, Lausitzer Seenland Klinikum GmbH, Hoyerswerda, Germany; ^11^Neurologische Uniklinik Ulm im RKU, Ulm, Germany; ^12^Fachklinik für Neurologie Dietenbronn, Schwendi, Germany; ^13^Department of Neurology, Charité – Universitätsmedizin Berlin, Corporate Member of Freie Universität Berlin, Humboldt-Universität zu Berlin, Berlin Institute of Health, Berlin, Germany

**Keywords:** multiple sclerosis, neuroprotection, safety, flupirtine, randomized controlled trail, hepatotoxicity

## Abstract

Central nervous system inflammation and neurodegeneration are the pathophysiological hallmarks of multiple sclerosis (MS). While inflammation can readily be targeted by current disease modifying drugs, neurodegeneration is by far less accessible to treatment. Based on suggested additional neuroprotective capacities of the orally available non-opioid and centrally acting analgesic drug flupirtine maleate we hypothesized that treatment with flupirtine maleate might be beneficial in MS patients. The *fl*upirtine as *or*al treatment *i*n *m*ultiple *s*clerosis (FLORIMS) study was a multi-center, randomized and stratified, placebo-controlled double-blind phase II trial to investigate safety and efficacy in terms of clinical and radiographical activity of flupirtine maleate (300 mg per day) given orally for 12 months, add-on to interferon beta 1b subcutaneously in patients with relapsing remitting MS. Due to a substantial delay in recruitment, enrolment of patients was prematurely terminated after randomization of only 30 of the originally planned 80 patients. Of these, 24 regularly terminated study after 12 months of treatment. Data were analyzed as originally planned. Treatment with flupirtine maleate was overall well tolerated. We observed moderate and asymptomatic elevations of liver enzymes in several cases but no overt hepatotoxicity. Neither the intention to treat nor the per protocol analysis revealed any significant treatment effects of flupirtine maleate with respect to occurrence of MS relapses, disability progression, or development of new lesions on cranial MRI. However, substantial methodological limitations need to be considered when interpreting these results. In conclusion, the results of the FLORIMS study neither add further evidence to nor argue against the hypothesized neuroprotective or disease modifying effects of flupirtine maleate in MS.

## Introduction

Neurodegeneration, i.e., damage to neuronal and axonal structures is a histopathological hallmark of multiple sclerosis (MS) (1), occurs already early in the course of disease (2), and is considered responsible for the development of irreversible neurological deficits (3, 4). Whether neurodegeneration in MS occurs exclusively secondary to an underlying autoimmune attack to central nervous system (CNS) structures or is in part a primary feature is a matter of debate. Beyond dispute is the perception that prevention of neurodegeneration is a highly important albeit largely unmet need in the treatment of MS (5).

Flupirtine maleate (hereafter referred to as flupirtine) is an orally available, non-opioid and centrally acting analgesic drug and is licensed in many European countries for the treatment of acute and chronic pain. Interestingly, it was never licensed in the United States. For decades, it was considered well tolerable without cancero- or teratogenic capacities and without a potential for tolerance and dependency even in long term use (6, 7). Nonetheless, potentially severe hepatotoxicity has been reported (8) which resulted in the confinement of indication in 2013. In 2018, the European Medical Agency (EMA) recommended withdrawal from the market.

Its main mode of action is stabilization of the neuronal membrane potential by selective opening of inwardly rectifying neuronal potassium channels. Flupirtine can cross the blood brain barrier (9, 10), and several lines of both *in vitro* and *in vivo* evidences from animal models suggest that flupirtine has neuroprotective properties (11–14). Furthermore, we have shown that flupirtine protects neurons from cytokine mediated death in a human living brain slice culture model (15). Also, a significantly increased survival of retinal ganglion cells and an improved visual function was observed in a rat model of autoimmune optic neuritis when animals were treated with flupirtine in addition to interferon beta (16). In a double blind placebo controlled trial in patients with Creutzfeld Jakob disease flupirtine treatment supported the preservation of cognitive functions (17).

Based on these promising data and long before the hepatotoxicity issue became evident we hypothesized that daily flupirtine intake prevents patients from MS-induced neuroaxonal damage and addressed this hypothesis in an investigator-initiated double blind randomized placebo-controlled interventional trial using clinical and magnetic resonance imaging (MRI) metrics as exploratory outcome parameters.

## Methods

### Study design and ethics statement

The *fl*upirtin as *or*al treatment *i*n *m*ultiple *s*clerosis (FLORIMS) study was designed as a multi-center, randomized and stratified, placebo-controlled, double-blind, explorative phase II trial. The aim was to investigate safety and efficacy in terms of clinical and MRI activity of flupirtine (final dose 100–0–200 mg per day) given orally for 12 months, add-on to interferon beta 1b (IFN β-1b) subcutaneously in patients with relapsing remitting (RR) MS. Originally planned was the enrolment of 80 RRMS patients (40 per study arm) at four German study centers (Berlin, Göttingen, Ulm, Cottbus, for details see Supplementary Material 1). Due to unforeseen difficulties in the recruitment of eligible participants, enrolment was prematurely terminated after inclusion of only 30 patients during a recruitment period of 60 months (December 2007–November 2012). The study was approved by the German Federal Institute for Drugs and Medical Devices (BfArM, 4032838), as well as by the local ethics committees (ZS EK 15510/06), and is registered at European Clinical Trials Database (EudraCT 2006-005262-39) and ClinicalTrials.gov (NCT00623415). It was conducted strictly adhering to the study protocol and to applicable national laws (Arzneimittelgesetz, 14. Novelle, 2005), the Harmonized Tripartite Guideline for Good Clinical Practice (ICH GCP) and the principles of the Declaration of Helsinki in its applicable version. All participants gave written informed consent at screening prior to any study-related procedures.

**Figure 1:**
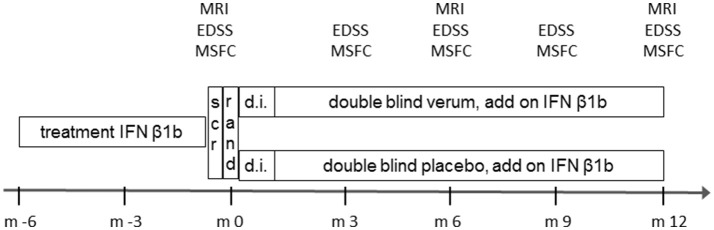
Design of the FLORIMS study. scr, screening; EDSS, expanded disability status scale; IFN, interferon; m, month; MRI, magnetic resonance imaging; MSFC, multiple sclerosis functional composite; rand, randomization.

The trial design is presented in Figure 1. After a short screening period eligible patients were stratified according to sex and T2 weighted (T2w) hyperintense lesion load in the baseline MRI and were then randomized 1:1 to either verum or placebo treatment add-on to continuing treatment with IFN β-1b. In the verum group, flupirtine was started at a dose of 100 mg once daily. After 2 weeks, the dose was increased to 100 mg twice daily, and after another 2 weeks to 100–0–200 mg. Treatment was continued for 12 months. Clinical evaluations including expanded disability status scale (EDSS) (18) and MS functional composite (MSFC) (19) were performed every 3 months, standardized MRI was performed at screening and after 6 and 12 months. Pharmacovigilance laboratory testing was done at least monthly in the first 6 months and thereafter every 3 months.

Designed as an explorative trial, no formal primary endpoint was defined in advance. For the same reason, no statistical sample size determination was accomplished.

All study participants had a minimum pretreatment of 6 months with IFN β1b. After a short screening period (1 week), patients were stratified and randomized 1:1 to receive either verum or placebo. The study drug dose was increased from 100 mg once daily for 2 weeks over 100 mg twice daily for the following 2 weeks to the final dose of 100–0–200 mg. Patients were kept on this dose for the next 11 months. Main investigations were performed at the time points displayed. Safety visits including vital signs and blood tests were done at months 0.5, 1, 1.5, 2, 3, 4, 5, 6, 9, and 12.

### Study population

The main inclusion criteria were: male and female patients with a definite diagnosis of MS according to the 2005 revised McDonald criteria (19) and a relapsing remitting disease course; age 18–55 years; EDSS score of 0–4 (18); continuous treatment with IFN β1b for at least 6 months. Important exclusion criteria were: preexisting liver disease or treatment with hepatotoxic drugs; any other CNS disease; pregnancy; inability to provide informed consent; incompatibility with MRI procedures. A comprehensive listing of in- and exclusion criteria is provided in the Supplementary Material 2.

### Outcome measures

All evaluations and scorings were performed by trained evaluators blinded to the patients' treatment allocations. Relapses were counted on the basis of generally accepted relapse definitions: (re)occurrence of new or previous CNS dysfunction in the absence of infections or hyperthermia, duration ≥24 h, time-lag from onset of previous relapse ≥30 days. MS-related disability was assessed by EDSS and MSFC (18, 19). EDSS scoring was performed by trained and certified (Neurostatus) physicians otherwise not involved in the management of study participants. MSFC scoring was done by trained study personnel according to standardized operating procedures. Standardized MRI scanning in all participants was done at the study center at Charité-Universitaetsmedizin Berlin using a 1.5 Tesla scanner (Siemens Medical Systems, Erlangen, Germany) as previously described (20). In short, an axial triple echo spin-echo sequence (TR 5,780 ms, TE1 13 ms, TE2 81 ms, TE3 121 ms, 3 mm slice thickness, 44 contiguous axial slices) was used to obtain proton density and T2w images. Additionally, we applied an axial fluid-attenuated inversion recovery sequence (TIRM, TR 10,000 ms, TE 108 ms, TI 2,500 ms, 3 mm slice thickness, 44 contiguous axial slices) and a sagittal high resolution 3-dimensional T1w sequence (MPRAGE, TR 2,110 ms, TE 4.38 ms, TI 1,100 ms, flip angle 15 degree, isotropic resolution 1 mm^3^). Conventional axial spin-echo T1w (TR 1,060 ms, TE 14 ms, 3 mm slice thickness, 44 contiguous axial slices) and axial T1w magnetization-prepared images (MTI, TR 1,290 ms, TE 14 ms, 3 mm slice thickness, 44 contiguous axial slices) were obtained before and 5 min after injection of 0.1 mmol/kg Gd-DTPA (Magnevist, Bayer-Schering, Berlin, Germany). An axial epi-planar (EPI) diffusion-weighted sequence (DWI, TR 9,400 ms, TE 118 ms, 3 mm slice thickness, matrix 1286128, b values 1 and 1,000 s/mm2) was acquired in 3 directions for the calculation of the ADC. Image quality was reviewed according to pre-determined criteria. Raw data were transferred to a Linux workstation and processed following a semi-automated procedure described previously (21), including an image coregistration (FMRIB's Linear Image Registration Tool, FMRIB Analysis Group, University of Oxford, Oxford, UK) and inhomogeneity correction routine embedded into the MedX v.3.4.3 software package (Sensor Systems Inc., Sterling, VA, USA). Bulk white matter lesion load and lesion count of T2w scans, as well as number and volume of contrast enhancing and hypointense lesions on T1w scans, were routinely measured using the MedX v.3.4.3 software package. Magnetization Transfer Ratio (MTR) was calculated in MIPAV (Medical Image Processing, Analysis, and Visualization, CIT-NIH, Bethesda, MD, USA) as previously described (22). MRI analyses were conducted in an anonymized way, applying a semi-automated procedure.

### Statistical analysis

Due to lack of data no statistical sample size calculation was performed in advance using a given error of the 1st kind and stipulated power. The chosen sample size of 80 patients (40 in each arm) was based mainly on practicability. Due to the explorative character of the study, statistical testing has to be understood as explorative, and data analyses were mainly descriptive for all endpoints. For univariate independent group comparisons exact Mann-Whitney-U tests and exact Chi-Square tests were used. For time series data, a nonparametric analysis of longitudinal data in a two-factorial design was applied (1st factor (independent): groups, 2nd factor (dependent): time). Statistical significance was assumed at the *p* = 0.05 level. Because of the explorative nature of analyses, no adjustments for multiple comparisons were performed. Both, intent-to-treat (ITT) and per-protocol (PP) analyses were carried out. The ITT group comprised 30 patients (15 verum; 15 placebo). The PP definition was regular study termination and mean study drug intake over the complete study duration of at least 85% of the per protocol scheduled intake. The PP group comprised 20 patients (11 verum; 9 placebo). All calculations were performed using SAS Version 9.4 [TS1M3] Copyright^©^ 2002–2012 by SAS Institute Inc., Cary, NC, USA, SPSS 24 (SPSS Inc., Chicago, IL, USA) and The R Project for Statistical Computing, Version 3.4.0 (2017-04-21), Copyright^©^ 2017.

## Results

**Figure 2:**
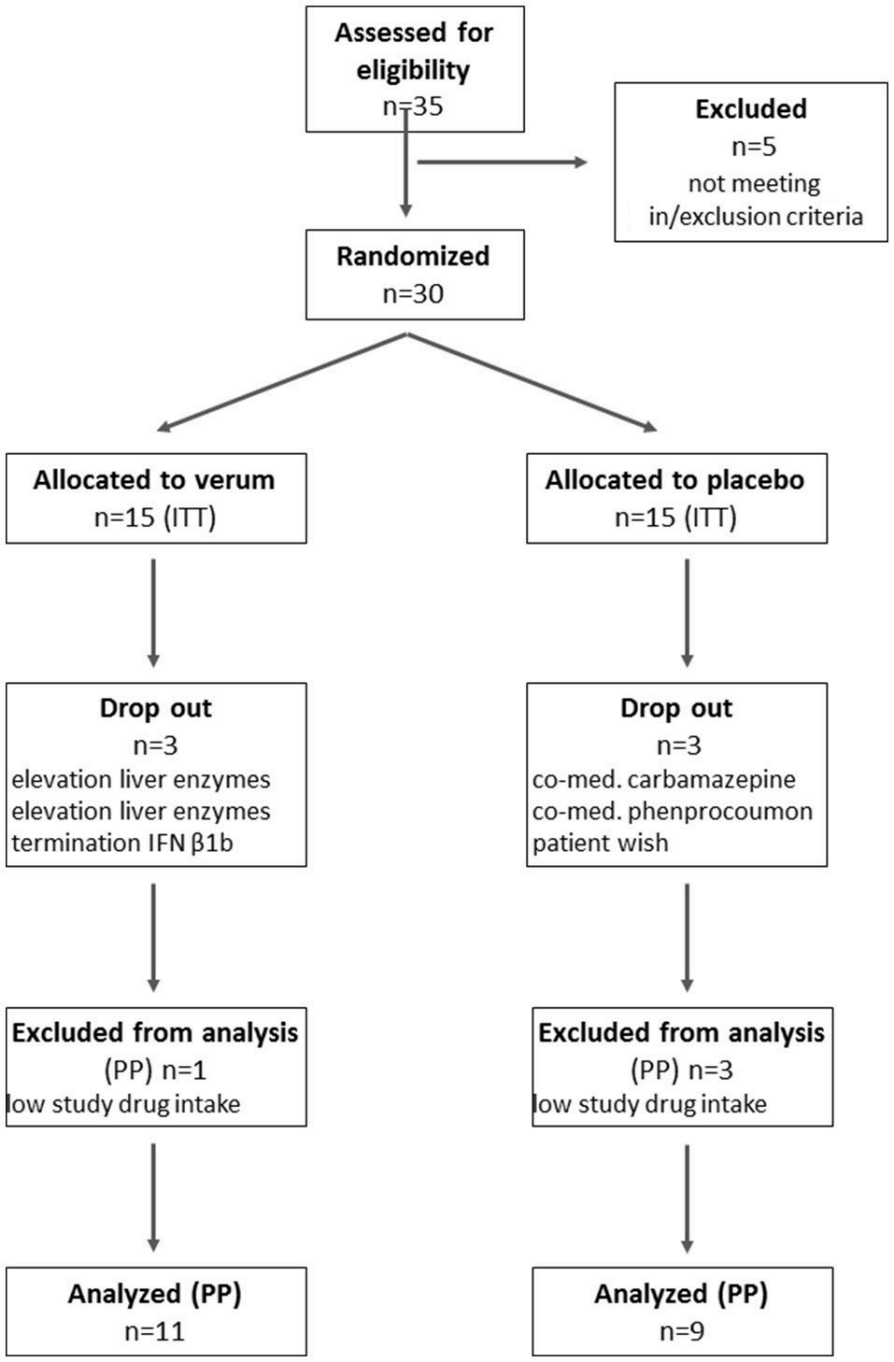
CONSORT flow diagram. ITT, intent to treat; PP, per protocol; *n*, number.

Of 35 MS patients screened for participation in the FLORIMS study, 30 patients were randomized and are included in the ITT analyses. Of these, six patients prematurely terminated study participation. The reasons for screening failure and premature termination are provided in the CONSORT flow diagram (Figure 2). The demographic characteristics of patients are presented in Table 1. There were no significant differences between the verum and placebo groups.

**Table 1:** Patients characteristics and baseline data.

	**Verum**	**Placebo**	
Total (*n*) [within group (%)]	15 [50]	15 [50]	n.s.^a^
Female [within group (%)]	8 [57]	6 [47]	n.s.^a^
Stratum lesion load high (*n*) [within group (%)]	10 [67]	12 [80]	n.s.^a^
Age (mean)	40 [2.0]	38 [2.8]	n.s.^b^
Mean MS-duration since onset (months) [SE]	115 [23]	122 [24]	n.s.^b^
Mean treatment IFN β1b (months) [SE]	60 [14]	52 [11]	n.s.^b^
BMI [SE]	26,8 [1,1]	28 [2,4]	n.s.^b^
EDSS (median) [range]	2.5 [1.5-4.0]	2.0 [0-4.0]	n.s.^b^
Patients with CEL [within group (%)]	0 [0]	3 [20]	n.s.^a^
Mean T2w lesion count (*n*) [SE]	29.4 [5.8]	27.6 [4.1]	n.s.^b^
Mean T2w lesion volume (mm^3^) [SE]	4298 [978]	4399 [1209]	n.s.^b^

*This table displays the most relevant characteristics of study participants at randomization. There were no significant differences between verum and placebo groups. BMI, body mass index; EDSS, expanded disability status scale; CEL, contrast enhancing lesions; n, number; n.s, not significant; SE, standard error*.

a *Exact Chi-Square tests*.

b *Exact Mann Whitney U-tests*.

Figure 2 shows the numbers of screened, randomized, and analyzed patients in the respective groups. The reasons for exclusion from randomization, drop out or exclusion from analysis are displayed.

### Clinical and mri endpoints

Important explorative clinical and MRI outcome parameters (univariate, ITT population) are presented in Table 2. In the verum group, nominal fewer patients had relapses, and the number of contrast enhancing lesions was lower. However, these differences were not significant. In summary, upon 12 months of continuous treatment with flupirtine, there were no significant differences in the occurrence of relapses, disability progression (EDSS, MSFC total, PASAT, TWT, 9HPT individually), occurrence of contrast enhancing lesions, increase in T2w lesion count and T2w lesion volume in comparison to placebo treatment. Additional longitudinal multivariate analyses of clinical parameters (EDSS, MSFC) also did not reveal a significant treatment effect of flupirtine (not shown). Results were comparable with those from the analysis of the PP population.

**Table 2:** Main clinical and MRI outcome parameters after 12 months.

	**Verum**	**Placebo**	
Patients with relapses (*n*)	2	6	n.s.^a^
Mean EDSS change [SE]	−0.25 [0.14]	+ 0.38 [0.25]	n.s.^b^
Mean PASAT change [SE]	+3.2 [2.0]	+10.7 [3.2]	n.s.^b^
Mean TWT z-score change [SE]	+0.8 [0.1]	+0.2 [0.16]	n.s.^b^
Mean 9HPT z-score change [SE]	+0.5 [0.2]	+0.1 [0.16]	n.s.^b^
Total patients with CEL (*n*)	1	5	n.s.^a^
Total number of new T2w lesions (*n*)	+6	+7	n.s.^b^
Mean T2w volume change (mm^3^)	+306	+828	n.s.^b^

*This table displays the most relevant outcome parameters after 12 months of double blind treatment. There were no significant differences between verum and placebo groups. EDSS, expanded disability status scale; CEL, contrast enhancing lesions; n, number; n.s, not significant; PASAT, paced auditory serial addition test; TWT, timed walk test; SE, standard error; 9HPT, 9-hole peg test*.

a *Exact Chi-Square tests*.

b *Exact Mann Whitney U-tests*.

### Safety endpoints

The study drug was overall well tolerated. No safety concerns were raised during the study. The number of documented adverse events (AE) was higher in the verum group (*n* = 90) than in the placebo group (*n* = 55), but this difference was not significant (exact Chi-Square, *p* = 0.54). The majority of AE was mild or moderate and not considered related to the study medication. Six cases of asymptomatically elevated liver enzymes were recorded, five in the verum group and one in the placebo group. Two of these, both in the verum group, prematurely terminated study participation. Two events in the verum group and one event in the placebo group were classified as severe (S) AE (bone fracture, pulmonary embolism, cholecystectomy). None of these was considered related to the study medication.

## Discussion

The FLORIMS study is the only trial so far that evaluated the efficacy of flupirtine on MS disease activity when given in addition to the standard disease modifying treatment IFN β1b. The rationale for this trial was provided by flupirtine's ability to cross the blood brain barrier (9, 10) and the demonstration of neuroprotective effects in both animals and humans (11–17). The study was carefully planned, and several efforts have been done to account for heterogeneity of patients and variability of data, e.g., stratification before randomization, monocentric MRI procedure for all patients, training for EDSS raters, strict separation of treating, and evaluating site personnel. We failed, however, to detect any significant effect of 12 months treatment on multiple exploratory clinical and MRI endpoints of disease activity. Besides a true lack of efficacy, several methodological issues need to be considered when interpreting these negative results. First to mention is the small sample size. Due to unforeseen substantial difficulties in the recruitment of initially targeted 80 participants, we had to prematurely terminate enrolment after randomization of 30 patients, of which only 24 completed the study on plan. Based on these numbers and a significance threshold of α = 0.05, the power to detect significant changes in EDSS was 59%. Twenty five patients per group would have allowed a power of 80%, suggesting that the initially planned 40 patients per group would have probably been sufficient. In light of the nominally lower numbers of both relapses and patients with contrast enhancing lesions as well as the milder increase of mean T2 hyperintense lesion volume in the verum group we cannot exclude that the lack of significance in these parameters is rather a consequence of poor power, i. e., that the negative outcome might reflect a type II error. Next, the number of relapses and new T2w or contrast enhancing lesions was rather low indicating a population with fairly low disease activity. This was probably at least in part attributable to the IFN β treatment, but a non add-on study design would have not been acceptable from an ethical point of view. Finally, the treatment and observation period of 12 months was probably not sufficient to evaluate the neuroprotective potential of the compound.

Importantly, despite asymptomatic elevation of liver enzymes in several cases we did not observe any clinically relevant hepatotoxicity within approximately 15 treatment years with 300 mg flupirtine per day which however will certainly not influence the EMA decision to withdraw flupirtine from the market.

In conclusion, the results of the FLORIMS study do not add further evidence for neuroprotective or disease modifying effects of flupirtine in MS, although in view of methodological limitations a false negative outcome, i.e., a type II error cannot be ruled out. Further research on flupirtine will however be hampered by its expected withdrawal from the market.

## Author contributions

All authors approved the final version of the manuscript. JD designed the trial, drafted the study protocol, treated patients, generated data, and drafted the manuscript. K-DW was the responsible biometrician, performed the statistical analyses, and drafted the manuscript. JW was in charge of acquisition and processing of MRI data and drafted the manuscript. JB-S treated patients and generated data. VS treated patients and generated data. MBS and MS were principal investigators at study center Göttingen, Germany, treated patients and generated data. AL was principal investigators at study center Cottbus, Germany, treated patients and generated data. HT was principal investigators at study center Ulm, Germany, treated patients and generated data. FP was initial principal investigators at study center Berlin, Germany, designed the trial, drafted the study protocol, generated data, and drafted the manuscript.

### Conflict of interest statement

JD received research support by Bayer and Novartis, travel support by Bayer, Novartis, Biogen, Merck Serono, and honoraria for lectures and advisory by Bayer, Novartis, Biogen, Merck Serono, Roche, Sanofi Genzyme. JW is CEO of MIAC AG Basel, Switzerland. He served on scientific advisory boards of Actelion, Biogen, Genzyme-Sanofi, Novartis, and Roche. He is or was supported by grants of the EU (Horizon2020), German Federal Ministeries of Education and Research (BMBF) and of Economic Affairs and Energy (BMWI). JB-S received travel grants and speaking fees from Bayer Healthcare, Biogen, Merck Serono, Sanofi-Aventis/Genzyme, Teva Pharmaceuticals, and Novartis. MS received support by Novartis, travel support by Bayer, Novartis, Merck Serono, and honoraria for lectures and advisory by Bayer, Biogen, Sanofi Genzyme. AL received travel support by Bayer, Biogen, Novartis, and honoraria for lectures by Biogen, Novartis. HT received funding for research projects, lectures, and travel from Bayer, Biogen, Genzyme, Fresenius, Merck, Mylan, Novartis, Roche, Siemens Health Diagnostics, Teva, and received research support from Hertie-Stiftung, BMBF, DMSG, University of Ulm and Landesstiftung BW. FP serves on the scientific advisory board for Novartis; received speaker honoraria and travel funding from Bayer, Novartis, Biogen Idec, Teva, Sanofi-Aventis/Genzyme, Merck Serono, Alexion, Chugai, MedImmune, and Shire; is an academic editor for PLoS ONE; is an associate editor for Neurology^®^; Neuroimmunology and Neuroinflammation; consulted for SanofiGenzyme, Biogen Idec, MedImmune, Shire, and Alexion; and received research support from Bayer, Novartis, Biogen Idec, Teva, Sanofi-Aventis/Genzyme, Alexion, Merck Serono, German Research Council, Werth Stiftung of the City of Cologne, German Ministry of Education and Research, Arthur Arnstein Stiftung Berlin, EU FP7 Framework Program, Arthur Arnstein Foundation Berlin, Guthy Jackson Charitable Foundation, and National Multiple Sclerosis of the USA. The remaining authors declare that the research was conducted in the absence of any commercial or financial relationships that could be construed as a potential conflict of interest.
