# Supplementary material for: Disease Modification in Multiple Sclerosis by Flupirtine—Results of a Randomized Placebo Controlled Phase II Trial
Source: Front Neurol. 2018 Oct 9;9:842. doi: 10.3389/fneur.2018.00842 (PMC6190842; doi:10.3389/fneur.2018.00842)
Supplement: Supplementary file 1 [file Data_Sheet_1.PDF]

Supplementary material 1:

List of participating study centers

NeuroCure Clinical Research Center, Charité – Universitätsmedizin Berlin, Charitéplatz 1, 10117 Berlin, Germany
Principal investigator: Prof. Dr. Friedemann Paul

Universitätsklinikum der Georg- August-Universität, Abtlg. für Neurologie, Robert-Koch Str. 40, 37075 Göttingen, Germany
Principal investigator: Dr. Muriel Sättler; Prof. Dr. Mikael Simons

Universitätsklinikum Ulm, Klinik für Neurologie, Oberer Eselsberg 45, 89081 Ulm, Germany
Principal investigator: Prof. Dr. med. Hayrettin Tumani

Carl- Thiem Klinikum Cottbus gGmbH, Klinik für Neurologie, Thiemstr. 111, 03048 Cottbus, Germany
Principal investigator: Dr. Andreas Linsa
