# Supplementary material for: Disease Modification in Multiple Sclerosis by Flupirtine—Results of a Randomized Placebo Controlled Phase II Trial
Source: Front Neurol. 2018 Oct 9;9:842. doi: 10.3389/fneur.2018.00842 (PMC6190842; doi:10.3389/fneur.2018.00842)
Supplement: Supplementary file 2 [file Data_Sheet_2.PDF]

Supplementary material 2:

Inclusion and exclusion criteria

Inclusion

- Written informed consent
- Age 18 to 55 (at randomization)
- multiple sclerosis according to revised McDonald criteria (2005)
- relapsing remitting disease course
- Expanded disability status scale (EDSS) ≤ 4,0
- Continuous treatment with interferon-β1b for at least six months (at randomization)
- Reliable contraception in women of childbearing potential (Pearl index <1)
- Negative pregnancy test at randomization

Exclusion

- Any other disease course than relapsing remitting
- any other disease that might better explain symptoms and signs in the patient
- any condition that might interfere with cranial MRI or other relevant examinations
- relevant chronic gastrointestinal (incl. neoplasia, peptic ulcer, Crohn's disease, ulcerous colitis, malassimilation syndrome), pulmonary (incl. tuberculosis, chronic obstructive pulmonary disease), infectious (incl. lues, borreliosis, HIV) and degenerative (inl. Alzheimer's disease, Parkinson's disease, Huntington's disease) disorders
- relevant liver disease (incl. neoplasia, chronic hepatitis, history of liver failure, cholestasis, ALAT, ASAT, GGT or bilirubin > 3x upper limit of normal)
- Renal or bone marrow dysfunction defined by hemoglobin < 8,5 g/dl, WBC < 2,5/nl platelets < 125/nl, creatinine > 180 µmol/l
- Myasthenia gravis
- history of allerdic reaction to gadolinium-DTPA
- history of allerdic reaction to flupirtine maleate.
- concomittant treatment with hepatotoxic drugs, sedative drugs (barbiturates, benzodiazepines, tricyclic antidepressants, anticonvulsants (carbamazepine, oxcarbazepine, primidone, gabapentine, pregabaline), analgesics (opioids, paracetamol), phenprocoumon
- Alcohol or illegal drug abuse
- pregnancy or lactation
- Participation in other interventional clinical trials within 6 months before or any time during the FLORIMS trial
- Previous treatment within the last six months with mitoxantrone, cyclophosphamide, cyclosporine, monoclonal antibodies, azathioprine, any other immunomodulatory or immunosuppressant drugs except interferon-β1b or methylprednisolone
- medical, psychiatric or other condition that relevantly limits the ability of the patient to provide informed consent, or to comply with the protocol.

- Lack of agreement with data protection guidelines
